# Solidified Tea

**Published:** 1883-01

**Authors:** 


SOLIDIFIED TEA.—One hundred grms. of ground sugar and 10 grms. starch sugar are boiled with the quantity of water required for solution, until the mass becomes tenacious, but yet remains transparent. After cooling, 50 grms. of tea previously mixed with 50 grms. of dry sugar, are added. The plastic mass is pressed into moulds, and when solidified forms the preserved tea.

